# Advances in epigenome-wide association studies for common diseases

**DOI:** 10.1016/j.molmed.2014.07.002

**Published:** 2014-10

**Authors:** Dirk S. Paul, Stephan Beck

**Affiliations:** UCL Cancer Institute, University College London, London, WC1E 6BT, UK

**Keywords:** GWAS, EWAS, DNA methylation, disease mechanism, biomarker

## Abstract

Epigenome-wide association studies (EWASs) provide a systematic approach to uncovering epigenetic variants underlying common diseases. Discoveries have shed light on novel molecular mechanisms of disease and enabled the application of epigenetic variants as biomarkers. Here, we highlight the recent advances in this emerging line of research and discuss key challenges for current and future studies.

Many common diseases in humans are mediated by genetic and environmental factors. Genome-wide association studies (GWASs) have been instrumental in identifying common genetic variants associated with a multitude of complex traits including common diseases. By contrast, the systematic assessment of epigenetic variation has lagged behind. Epigenetic modifications of DNA and histone proteins are heritable (re-established) during cell division and can induce stable changes in the regulation of gene expression. Technological advances in high-throughput DNA analysis have facilitated the genome-wide examination of epigenetic modifications, primarily DNA methylation, enabling systematic, large-scale association testing with disease phenotypes 1, 2. Publications of such epigenome-wide association studies (EWASs) have soared in recent years, revealing novel molecular mechanisms of disease (akin to GWASs) but also substantial challenges in the interpretation of tentative associations. Below, we discuss what we consider to be the key issues concerning the assessment of EWAS findings: cellular heterogeneity, causal inference, and replication of identified DNA methylation variable positions (MVPs).

Global epigenetic patterns, such as the genome-wide distribution of DNA methylation marks, vary substantially across different cell lineages and cell types. Exogenous (e.g., smoking, diet, and medication) and endogenous (e.g., senescence) factors, as well as pathogenic (e.g., inflammatory) conditions, have an effect on cellular composition. Therefore, EWASs depend on analytical tools that determine epigenetic variation with respect to both confounding cellular heterogeneity and phenotype of interest. The importance of adjusting for the proportions of different cell populations was emphasised by Liu *et al.* [3] in an EWAS for rheumatoid arthritis, a common autoimmune disease. The authors probed DNA methylation marks in whole blood. Indeed, whole blood has proven to be the tissue of choice for most EWASs owing to its ease of accessibility. Importantly, they found that the proportions of the major circulating leukocytes differ between cases and controls. Statistical methods are capable of inferring and correcting for such cellular heterogeneity, either with [4] or without 5, 6 the use of reference data sets. Following reference-based adjustment, Liu *et al.* achieved a substantial reduction of spurious association signals attributed to cellular heterogeneity. Conversely, the detected changes in cell type composition based on global epigenetic patterns between cases and controls may in some studies delineate disease pathogenesis or progression itself, thereby informing biomarker discovery (Figure 1).

**Figure 1:**
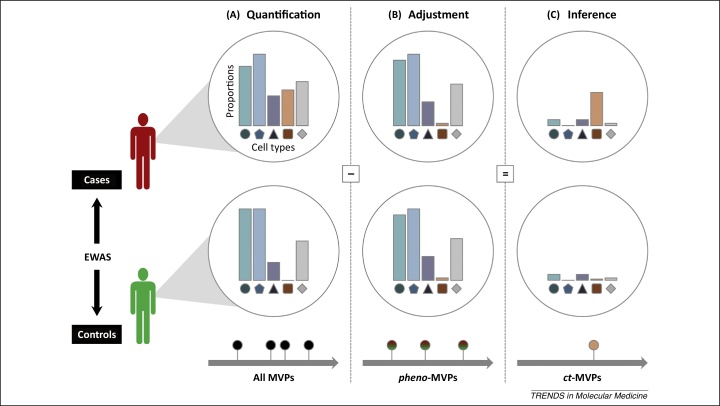
The relevance of cellular heterogeneity in EWASs. Statistical methods that assess and adjust for the proportions of different cell populations derived from the DNA methylation profile of a cell have emerged as powerful tools in epigenome-wide association studies (EWASs). Some algorithms do not depend on reference data sets 5, 6, whereas other algorithms use reference data sets consisting of major leukocyte cell types [4]. Additional data sets will likely be integrated once released by the International Human Epigenome Consortium (http://ihec-epigenomes.org/). In general, the use of reference data sets is preferred because the cellular composition of a sample can be estimated more accurately. Below, we distinguish between three types of DNA methylation variable positions (MVPs), all of which are informative in different contexts. **(A)** Without correction of cellular heterogeneity, identified DNA methylation changes between cases and controls inform the overall heterogeneity of the phenotype of interest (irrespective of the underlying source and mechanism). **(B)** If adjustment for confounding cellular heterogeneity is performed, identified MVPs most accurately reflect methylation changes relevant to the phenotype. Such *pheno*-MVPs can subsequently be grouped into functionally more relevant differentially methylated regions (DMRs). **(C)** The contrast of all MVPs identified (A) with *pheno*-MVPs (B) may further characterise the disease state. For example, in addition to epigenetic changes that are related to the phenotype, an increase in lymphocyte populations may offer valuable clues to the immunobiology of the disease state [4]. Indeed, MVPs attributed to differential cell type distribution (termed *ct*-MVPs) may also shed light on the potential involvement of additional (or thus far disregarded) cell types in disease pathogenesis.

Epigenetic variation can contribute to the development of a disease or be a consequence of it (also known as reverse causality). Distinguishing between the two processes presents a major challenge for EWASs. In an early EWAS for type I diabetes, another common autoimmune disease, a longitudinal cohort consisting of individuals that were profiled both before and after disease diagnosis and treatment was analysed [7]. Rakyan *et al.* found evidence of DNA methylation changes that precede clinical diagnosis, potentially contributing to the aetiology of overt type I diabetes. However, large prospective cohorts that enable the assessment of temporal origins of epigenetic changes are scarce, highlighting the need for alternative approaches. To this end, Dick *et al*. [8] supplemented their EWAS for body mass index (BMI), a measure of obesity, with a methylation quantitative trait loci (metQTL) analysis. This assessed the possibility of the identified MVPs being driven by genetic variants. The study identified BMI-associated MVPs at the *HIF3A* gene locus, and the most significant MVP was found to have a larger impact on BMI than previously reported genetic associations at the well-studied *FTO* gene locus. Two genetic variants were identified upstream of *HIF3A* that showed an independent effect on DNA methylation levels. However, these genetic variants were not associated with BMI, suggesting that the increased DNA methylation levels at the locus are likely to be a consequence of increased BMI rather than a cause. The finding that these MVPs were associated with BMI in subcutaneous adipose but not skin tissue further strengthened this notion. Nonetheless, the identified non-causal epigenetic changes may still be meaningful as diagnostic or prognostic biomarkers. In addition to metQTL analysis, causal dependency can also be assessed by Mendelian randomisation that has been specifically adapted for EWAS [11].

The aforementioned EWASs for rheumatoid arthritis [3] and BMI [8] stand out due to the sequential replication of the significant MVPs, which involved large numbers of samples and independent cohorts. In recent EWASs, the discovery cohort has usually been profiled using the Illumina Infinium HumanMethylation450 BeadChip (‘450K array’), an array-based platform that can probe more than 485,000 DNA methylation marks across the genome. By contrast, the platforms selected for replication vary but are usually low-throughput. As the field matures, analogous to GWASs, 450K array data sets will be generated for different sample cohorts of the same disease, empowering collaborative replication efforts and meta-analyses of EWASs. Alternatively, methylated DNA immunoprecipitation followed by deep sequencing (MeDIP-seq) offers a higher-density approach, capturing disease-relevant DNA methylation changes not interrogated by the sparse 450K array [9]. This approach is currently being applied to a large cohort of 5,000 twins discordant for various phenotypes (http://www.epitwin.eu/).

The study by Dick *et al.* [8] as well as an EWAS for multiple sclerosis, an inflammatory disease of the central nervous system [10], explored the potential functional impact of the identified epigenetic changes. Huynh *et al.* [10] performed RNA-sequencing in brain samples collected from multiple sclerosis patients and healthy controls. The authors identified genes with coordinated variation in DNA methylation and gene expression, following the dogma of reduced methylation at gene promoters and increased gene expression, and vice versa. Looking ahead, epigenetic changes will likely be functionally tested using the much-publicised genome-editing tools CRISPR/Cas9 and TALEN. Generally, such follow-up experiments add confidence and interpretability to the MVPs discovered through EWASs.

Taken together, in EWASs (and in stark contrast to GWASs) we advise caution with respect to the cellular heterogeneity of sample material and causal inference of the identified epigenetic changes. Nevertheless, we propose that an EWAS is a valuable approach for uncovering novel molecular mechanisms of disease, and identifying diagnostic and prognostic biomarkers.
